# Tertiary Lymphoid Structure in Dental Pulp: The Role in Combating Bacterial Infections

**DOI:** 10.1002/advs.202406684

**Published:** 2024-10-28

**Authors:** Ruiqi Li, Fan Gu, Linlin Peng, Tingting Huan, Zhuo Zhou, Yaling Song, Jinmei He, Kaili Ye, Yao Sun, Tiejun Li, Miao He, Zhuan Bian, Wei Yin

**Affiliations:** ^1^ State Key Laboratory of Oral & Maxillofacial Reconstruction and Regeneration Key Laboratory of Oral Biomedicine Ministry of Education Hubei Key Laboratory of Stomatology School & Hospital of Stomatology Wuhan University Wuhan 430079 China; ^2^ Department of Cariology and Endodontics (I) Hospital of Stomatology Wuhan University Wuhan 430079 China; ^3^ Department of Cariology and Endodontics (II) Hospital of Stomatology Wuhan University Wuhan 430079 China; ^4^ Department of Implantology Shanghai Engineering Research Center of Tooth Restoration and Regeneration Stomatological Hospital and Dental School of Tongji University Shanghai 200092 China; ^5^ Department of Oral Pathology National Center of Stomatology National Clinical Research Center for Oral Diseases National Engineering Laboratory for Digital and Material Technology of Stomatology Beijing Key Laboratory of Digital Stomatology Research Center of Engineering and Technology for Computerized Dentistry Ministry of Health NMPA Key Laboratory for Dental Materials Peking University School and Hospital of Stomatology Beijing 100081 China

**Keywords:** caries, chemokines, dental pulp, immune cells, pulpitis, tertiary lymphoid structure (TLS)

## Abstract

Tertiary lymphoid structure (TLS) is associated with various pathologies, including those of cancers and chronic infections. Depending on the organ, multiple factors regulate the formation of TLS. However, the role of TLS in immune response and the molecules that drive its formation remain uncertain. The dental pulp, includes a few immune cells surrounded by rigid mineralized tissue, and opens to the outside through the apical foramen. Owing to this special organization, the dental pulp generates a directional immune response to bacterial infection. Considering this aspect, the dental pulp is an ideal model for comprehensively studying the TLS. In the present study, single‐cell RNA sequencing of healthy and inflamed human dental pulp reveals known markers of TLS, including C‐C motif chemokine ligand 19 (CCL19), lysosome‐associated membrane glycoprotein 3 (LAMP3), CC chemokine receptor 7 (CCR7), and CD86, present in inflamed dental pulp. Compared with the healthy pulp, types and proportions of immune cells increase, along with enhanced cellular communication. Multiple immunofluorescence staining reveals that typical TLS emerges in dental pulp with pulpitis, consistent with the high expression of CC chemokine ligand 3 (CCL3), which may be a key driver of TLS formation. Moreover, TLS is also observed in a mouse model of pulpitis. These findings collectively offer insights into the formation and function of TLS in response to infection.

## Graphical abstract

TLS is involved in the immune responses to cancer and other chronic inflammatory conditions, often associated with favorable clinical outcomes. In this context, dental pulp serves as an ideal model for chronic infection, and is used here to elucidate the specific contributions of TLS to the immune response and to identify the drivers of its formation.

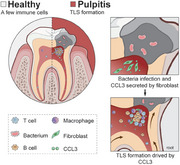

## 1 Introduction

The adverse effects on tissues and organs resulting from incomplete eradication of infecting bacteria are well documented. Fortunately, the immune system protects the body from these infections; and the lymphatic system, a constituent of the immune system, plays crucial roles in immunity. Dendritic cells (DCs) detect infecting bacteria and promptly migrate to adjacent lymph nodes.^[^ ^1^ ^]^ Within the lymph nodes, the effector T cells (under the supervision of regulatory T cells) inspect the surfaces of DCs for antigens and dispatch T cells that can recognize cognate antigens, thereby mounting a rapid immune response.^[^ ^2^ ^]^ Moreover, recent studies have demonstrated that, in response to prolonged and severe bacterial invasion, immune cells, including B cells, T cells, and macrophages, also develop a tertiary lymphoid structure (TLS) in non‐lymphoid tissues. This structure resembles secondary lymphoid organs but lacks capsules and is crucial in combating infection.^[^ ^3^, ^4^ ^]^ TLS has been observed in the invasive margins, stroma, and/or core of several tumor subtypes.^[^ ^5^, ^6^ ^]^ Because TLS promotes immune cell infiltration into solid tumors, its presence is frequently associated with improved patient survival.^[^ ^7^, ^8^, ^9^ ^]^ However, it is associated with an increased risk of advanced recurrence in hepatocellular carcinoma.^[^ ^10^ ^]^

Although the development of TLS is strongly associated with the progression and prognosis of various diseases,^[^ ^11^, ^12^ ^]^ its specific contribution to the immune response and the drivers of its formation remain uncertain. To address this gap in knowledge, the best model is a tissue that is initially devoid of immune cells and gradually recruits immune cells in response to infection.

To the best of our knowledge, the dental pulp is an ideal model for the following reasons: 1) healthy pulp is devoid of B cells, but includes small numbers of macrophages and T cells that act as an immune surveillance system;^[^ ^13^ ^]^ 2) the dental pulp is surrounded by dentin, and mainly comprises pulp cells and odontoblasts.^[^ ^14^, ^15^ ^]^ The apical foramen at the root tip of the tooth serves as the sole connection of the pulp to the external environment and thereby the only source of immune cells.^[^ ^16^ ^]^ Therefore, observing the immune response of dental pulp to bacterial invasion will provide insights into the formation and progression of TLS. In this study, we summarize the contributions of TLS to the immune response against bacterial invasion by examining the immune cell composition of the dental pulp at various stages of bacterial infiltration.

## 2 Results and Discussion

### 2.1 Cells Involved in the Formation of TLS

**Figure 1:**
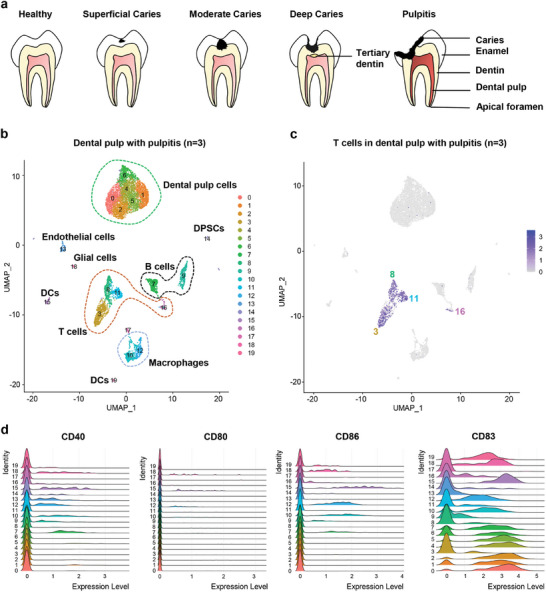
Cellular composition of freshly isolated human inflammatory dental pulp. a) Schematic diagram depicting the bacterial invasion process in the dental pulp. b) Uniform Manifold Approximation and Projection (UMAP) visualization of the inflammatory dental pulp (n = 3), colored by clusters. c) T cell clusters (cluster 3: cytotoxic T cells, cluster 8: Treg cells, cluster 11: Th cells, and cluster 16: Trm cells) in inflammatory pulp. Cells with high or low expression were marked with purple or gray color respectively. d) The expression of CD40, CD80, CD86 and CD83 in cluster 15 and 19.

Innumerable bacteria reside in the oral cavity. Several factors, including the utilization of glucose by acid‐producing bacteria, can lead to caries if not addressed promptly. Should the lesion remain untreated, bacteria may enter the pulp through the dentinal tubules, causing inflammation of the dental pulp (**Figure** **1**a).^[^ ^17^, ^18^ ^]^ To learn more about the changes in cellular component of the infected dental pulp, we performed single‐cell RNA sequencing (scRNA‐seq) on dental pulp extracting from the third molar which was in healthy or inflammation state. Consistent with our previous study,^[^ ^19^ ^]^ we did not limit the ratio of mitochondria, as cell viability was affected by the anesthetic injected before tooth extraction. Twenty cell clusters were identified in the inflamed pulp (Figure 1b; Figure S1a, Supporting Information), including pulp cells (clusters 0, 1, 2, 4, 5, and 6), T cells (clusters 3, 8, 11, and 16), macrophages (clusters 10 and 12), memory B cells (cluster 7), plasma cells (cluster 9), DCs (clusters 15 and 19), dental pulp stem cells (DPSCs) (cluster 14), glial cells (cluster 18), endothelial cells (cluster 13), and cells that expressed several mitochondrial genes as markers (cluster 17). Compared with normal pulp, the proportion of T cells (clusters 3, 8, 11, and 16) was significantly increased in inflamed dental pulp. Cluster 3 comprised cytotoxic T cells that expressed Eomesodermin (EOMES) and interferon‐gamma (IFNG). Cluster 8 comprised regulatory T (Treg) cells that express CD4, interleukin‐7 receptor (IL7R), and transcription factor forkhead box protein 3 (FOXP3). Cluster 11 comprised T helper (Th) cells that express inducible T‐cell costimulator (ICOS), CC chemokine receptor 6 (CCR6), and killer cell lectin‐like receptor subfamily B member 1 (KLRB1). Cluster 16 comprised tissue‐resident memory T (Trm) cells that express mini‐chromosome maintenance complex component 4 (MCM4) and cyclin‐A2 (CCNA2) (Figure 1c; Figure S1b, Supporting Information).

Clusters 15 and 19 comprised mature DCs (Figure 1d). Notably, cluster 15 cells expressed C‐C motif chemokine ligand 19 (CCL19), lysosome‐associated membrane glycoprotein 3 (LAMP3), CC chemokine receptor 7 (CCR7), and CD86, which are known markers of TLS.^[^ ^7^ ^]^ This finding prompted us to explore the presence of TLS in pulpitis and study its contribution to the immune response against bacteria (Figure S1c, Supporting Information).

Clusters 10 and 12 comprised M2 macrophages (Figure S1d, Supporting Information). Cluster 12 cells expressed the macrophage activity regulator, MER tyrosine kinase (MERTK),^[^ ^20^ ^]^ as well as macrophage‐polarizing factors such as interferon regulatory factor 5 (IRF5) and TGF‐beta‐induced factor homeobox 1 (TGIF1),^[^ ^21^ ^]^ whereas cluster 10 cells expressed versican (VCAN), epiregulin (EREG), and ficolin‐1 (FCN1).

Clusters 7 and 9 comprised memory B cells and plasma cells, respectively. Cluster 13 (endothelial cells) expressed the hematopoietic stem cell marker (CD34), endothelial markers [Cadherin 5 (CDH5) and kinase insert domain‐containing receptor (KDR)], ecto‐5′‐nucleotidase (NT5E), thymocyte differentiation antigen 1 (THY1), and key proteins of the Wnt and Notch signaling pathways (Figures S1e and S2b,c, Supporting Information). The endothelial cells in cluster 13 expressed 15 regulons, including SP2 (Figure S2a, Supporting Information).

### 2.2 TLS Recruits Immune Cells as Early as Possible

**Figure 2:**
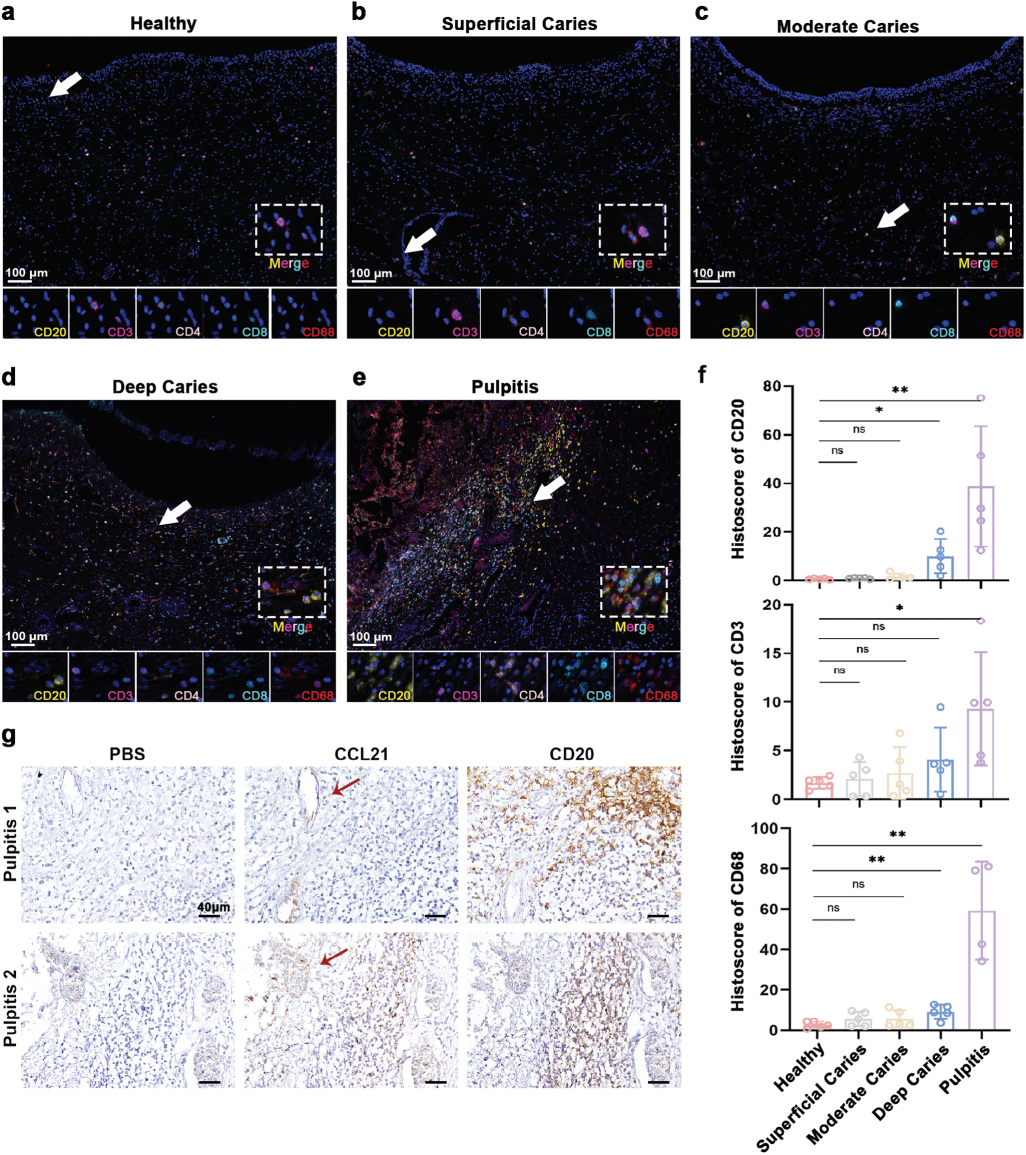
Multiplex immunofluorescence (mIF) staining and immunohistochemical (IHC) staining of TLS in human dental pulp. a–e) Distribution of CD20^+^ cells, CD3^+^ cells, CD4^+^ cells, CD8^+^ cells, and CD68^+^ cells in the dental pulp of different clinical samples (healthy tooth, tooth with superficial caries, moderate caries, deep caries and pulpitis). Red (CD68), yellow (CD20), pink (CD4), cyan (CD8), magenta (CD3), and blue (DAPI). White arrows and boxes represented the magnified areas. Scale bars = 100 µm. f) Histologic evaluation of the expression of CD20, CD3 and CD68 in dental pulp with healthy state (n = 5), or with superficial caries (n = 5), moderate caries (*n* = 5), deep caries (n = 5) and pulpitis (*n* = 5). g) Distribution of CCL21 in two pulpitis samples (Pulpitis 1 and 2). The area indicated by the red arrow represented the CCL21 expression. Scale bars = 40 µm. Two‐tailed unpaired Student's t‐test was used for statistical analysis. ns, no significant; ^*^ *p* < 0.05; ^**^ *p* < 0.01.

To accurately observe the TLS formation, we collected additional samples of healthy pulp and pulp with superficial caries, moderate caries, deep caries, and pulpitis (**Figure** **2**a–e; Figure S3, Supporting Information), and observed typical TLS in pulpitis using multiple immunofluorescence (mIF) staining (Figure 2e). Consistent with our previous results from the sequencing of healthy dental pulp,^[^ ^19^ ^]^ we observed no B cells in the healthy pulp (Figure 2a). Multiplex immunofluorescence imaging of healthy pulp and pulp with superficial caries revealed macrophages and T cells scattered throughout the pulp (Figure 2a,b). The dental pulp of the tooth with moderate caries (tooth where bacteria had begun to penetrate dentin) comprised few B cells (Figure 2c) and marginally increased numbers of T cells and macrophages close to the lesion sites‐that is, in the crown than in the root. These results suggest that the dental pulp begins recruiting immune cells well before the bacteria invade it. Prior to the formation of deep caries, the odontoblasts initiate the formation of tertiary dentin to increase the distance between the invading bacteria and dental pulp. By the time deep caries was reached, the number of B cells in the dental pulp were increased (Figure 2d). A significantly increased number of immune cells marked the progression of the disease to pulpitis (Figure 2e,f; Figure S4, Supporting Information). Considering these findings, we inferred that TLS recruited immune cells during the early stages of infection, where the bacteria were further away from the pulp.

The structure of TLS that we observed in pulpitis was consistent with the classical structure of TLS,^[^ ^22^ ^]^ wherein the B cells were located at the center and surrounded by T cells. Macrophages were scattered throughout the TLS. In addition, special blood vessels called high endothelial venules (HEVs) fed into the TLS, which facilitated transmigration of lymphocytes from the circulation into lymphoid tissues.^[^ ^23^ ^]^ The endothelial cells of HEVs expressed CC chemokine ligand 21 (CCL21), a ligand of the chemokine receptor CCR7, thereby aiding in lymph node homing (Figure 2g).^[^ ^24^ ^]^ We inferred that TLS was formed to combat the infection, and HEVs were necessary to recruit lymphocytes.

### 2.3 TLS‐Associated Factors in Inflammatory Dental Pulp

**Figure 3:**
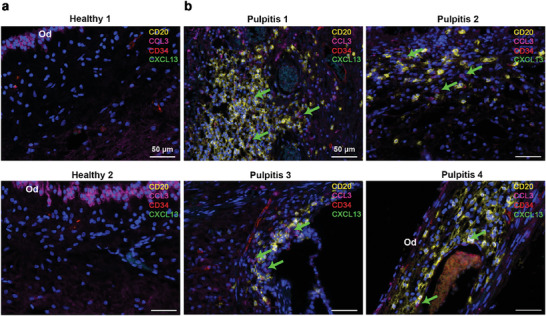
Multiplex immunofluorescence staining of TLS in human dental pulp. a) Distribution of CD20^+^ cells, CD34^+^ cells, CCL3^+^ cells, and CXCL13^+^ cells in healthy dental pulp (Healthy 1 and 2). Colors were indicated as follows: Red (CD34), Yellow (CD20), Magenta (CCL3), Green (CXCL13), and Blue (DAPI). Od, odontoblast. Scale bars = 50 µm. b) Distribution of TLS marked by CD20^+^ cells, CD34^+^ cells, CCL3^+^ cells, and CXCL13^+^ cells in inflamed dental pulp (Pulpitis 1, 2, 3, and 4). Color coding as above. The area indicated by the green arrow represented the CCL3 expression. Od, odontoblast. Scale bars = 50 µm.

Chemokines play important roles in inducing TLS formation.^[^ ^25^ ^]^ Therefore, we analyzed the expression levels of several chemokines. The expression levels of the three major chemokines that induce TLS formation, including CCL19, CCL21, and C‐X‐C motif chemokine ligand 13 (CXCL13),^[^ ^26^ ^]^ were low in most cells of the dental pulp; a small number of T cells (clusters 11 and 16) expressed CXCL13. In contrast, we diverted our attention to the elevated expression of CC chemokine ligand 3 (CCL3). To confirm the role of CCL3 in the formation of TLS, we performed mIF staining. The results consistently showed that changes in immune cells were accompanied by alterations in CCL3 expression. In healthy dental pulp, CCL3 is predominantly expressed in odontoblasts and, to a lesser extent, in pulp cells (**Figure** **3**a). In pulpitis, CCL3 was notably present at the periphery of immune cells (Figure 3b), suggesting that CCL3 might be a critical driver of TLS formation. In humans, CCL3 receptors include insulin‐degrading enzyme (IDE), atypical chemokine receptor 2 (ACKR2), CC Chemokine receptor 1 (CCR1), CC chemokine receptor 5 (CCR5), and CC chemokine receptor 9 (CCR9).^[^ ^27^, ^28^ ^]^ Immune cells in inflamed pulp expressed more IDE than non‐immune cells. Therefore, we hypothesized that CCL3‐IDE was a key factor in the formation of TLS.

### 2.4 TLS Maximize the Function of Immune Cells

**Figure 4:**
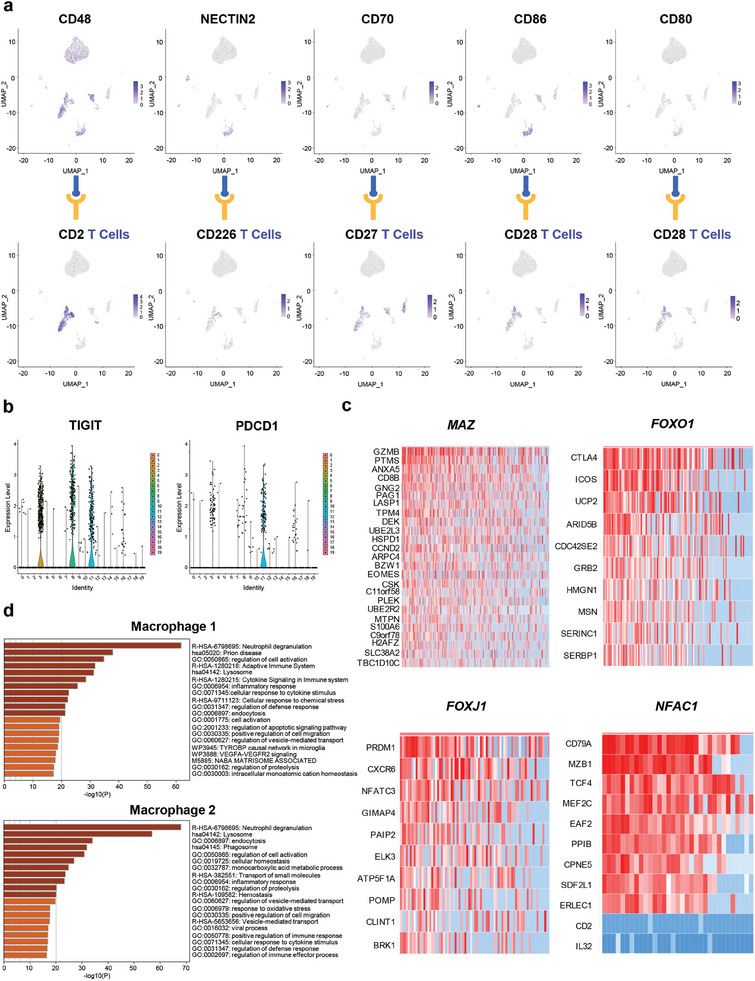
Activation and cross‐talk of T cells and other cells in TLS. a) Distribution of co‐stimulatory molecules of T cells (CD2, CD226, CD27, and CD28), and their ligands (CD48, NECTIN2, CD70, CD86, and CD80) in cells from inflamed pulp. b) The ligands of co‐inhibitors of T cells (TIGIT and PDCD1) distributed in macrophages, DCs and pulp cells. c) The regulons of four T cell types (*MAZ*, the mainly regulon of cytotoxic T cells; *FOXO1*, the mainly regulon of Treg cells; *FOXJ1*, the mainly regulon of Th cells; *NFAC1*, the mainly regulon of Trm cells). d) Molecular functions of unique marker genes in macrophage 1 (up) and macrophage 2 (down).

Immune cells aggregate in the dental pulp to eliminate the invading bacteria. Therefore, we analyzed the functions and intercellular communication of these immune cells. Our scRNA‐seq data indicated that several co‐stimulatory molecules, including CD2, CD27, CD28, and CD226, were expressed by four T‐cell clusters; moreover, their ligands were distributed in macrophages and DCs (**Figure** **4**a). The ligands of the co‐inhibitory molecules, including T cell immunoreceptor with Ig and immunoreceptor tyrosine‐based inhibitory domains (TIGIT) and programmed cell death 1 (PDCD1), were mainly distributed in macrophages, DCs, and pulp cells (Figure 4b). Regulons in T cells included MYC‐associated zinc‐finger protein (*MAZ)*, Forkhead box protein O1 (*FOXO1)*, Forkhead box protein J1 (*FOXJ1)*, and Nuclear factor of activated T‐cells, cytoplasmic 1 (*NFAC1)* (Figure 4c). Several critical activities, including lymphocyte activation, regulation of lymphocyte activation, and alpha‐beta T cell activation, suggested active T cell function in the inflamed pulp (Figure S5a, Supporting Information).

CD2 was the primary ligand of T cells exhibiting the most interactions with other cell types, while Tregs demonstrated minimal contact. CD8^+^ T cells generally showed limited communication with other cell types; however, an increase in interactions with both DPSCs and pulp cells in pulpitis was noted compared to the normal state. The communication between T cells and DPSCs was mediated by tumor necrosis factor (TNF) and tumor necrosis factor ligand superfamily member 12 (TNFSF12), while interactions between T cells and endothelial cells involved macrophage colony‐stimulating factor 1 (CSF1), receptor‐type tyrosine‐protein phosphatase C (PTPRC), tumor necrosis factor ligand superfamily member 10 (TNFSF10), and L‐selectin (SELL). Additionally, galectin‐9 (LGALS9), TNF, CD40 ligand (CD40LG), and interleukin‐1 receptor (IL‐1R) facilitated communication between T cells and pulp cells. Signals among T cells were transmitted via CSF1, tumor necrosis factor ligand superfamily member 14 (TNFSF14), and neurogenic locus notch homolog protein 1 (NOTCH1) (Figure S5b, Supporting Information).

Despite significant differences in ligand‐receptor pairs on the surfaces of the two macrophage clusters, macrophage communication was notably enhanced compared to the normal state, particularly with endothelial cells. Macrophages recruited endothelial cells using chemokines [CCL3/4/5 and C‐X‐C motif chemokine 5 (CXCL5)], transmitted signals via TNF and delta‐like protein 1 (DLL1), and promoted angiogenesis through sphingosine‐1‐phosphate phosphatase 1 (SPP1), TNFSF12, and tumor necrosis factor ligand superfamily member 15 (TNFSF15). They also received signals from ephrin‐B1 (EFNB1), ephrin‐B2 (EFNB2), and tumor necrosis factor receptor superfamily member 10A (TNFRSF10A) in endothelial cells along with ephrin type‐B receptor 2 (EPHB2). Macrophage communication was mediated not only by chemokines and their receptors but also by CSF1, nicotinamide phosphoribosyltransferase(NAMPT), neuregulin‐1 (NRG1), CD226, and CD80. Interactions between macrophages and T cells involved chemokines and cell adhesion‐related factors, with macrophages sending activation signals to T cells via CSF1, ICOS ligand (ICOSLG), interleukin‐15 (IL15), and TNF, and receiving signals from T cells through adenosine receptor A3 (ADORA3), tumor necrosis factor receptor superfamily member 3 (LTBR), and TNFRSF10A/B (Figure S5c, Supporting Information). Macrophages in cluster 10 were immunologically more active than those in cluster 12 (Figure 4d). Ribosomes were active in B cells, aligning with cell cycle analysis (Figure S5d, Supporting Information).

### 2.5 TLS Timely Preserves Viable Cells in Case of Severe Bacterial Infections

**Figure 5:**
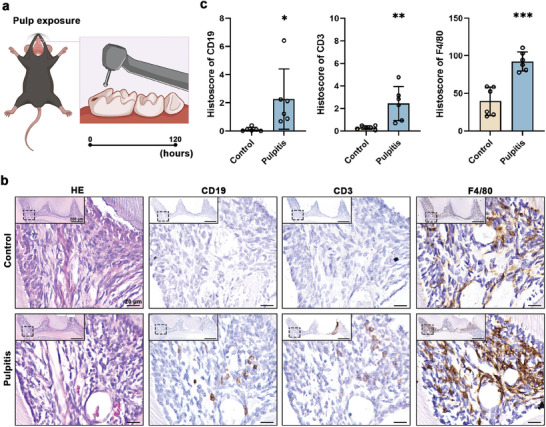
The occurrence of TLS in mouse pulpitis. a) The diagram of the mouse pulpitis model construction. b) Hematoxylin and eosin (H&E) staining  and IHC staining of CD19, CD3 and F4/80 in serial sections of mouse pulpitis (*n* = 6). The high magnification boxes represented the areas in dotted boxes. Scale bars = 200 or 20 µm. c) Histologic evaluation of CD19, CD3, and F4/80 expression in experimental pulpitis (*n* = 6). Two‐tailed unpaired Student's *t*‐test was used for statistical analysis. ^*^ *p* < 0.05, ^**^ *p* < 0.01, ^***^ *p* < 0.001.

To further validate these results, we developed experimental murine models of pulpitis (**Figure** **5**a). The results depicted in Figure 5b indicate a similar situation in mouse dental pulp. Immunohistochemistry (IHC) staining of CD19 (a B cell marker), CD3 (a T‐cell marker), and F4/80 (a macrophage marker) in serial sections revealed colocalization, suggesting the potential presence of TLS in mouse pulpitis (Figure 5b). In addition, TLS were observed at the forefront of pulpitis lesions in mice, which is consistent with the results in human pulpitis. This suggests that TLS may act as a defensive barrier against bacterial invasion. The expression levels of CD19, CD3, and F4/80 were elevated in the experimental pulpitis of mice compared to those in the control group, as shown in Figure 5c.

## 3 Conclusion

In summary, in the dental pulp with pulpitis, TLS prompted the recruitment of immune cells and increased communication between immune cells and other cell types. The TLS fought against bacterial infections at the forefront of inflamed dental pulp.

## 4 Experimental Section

### Materials

Primary antibodies, Anti‐CD3 (ab213362), Anti‐CD19 (ab245235), Anti‐CD3 (ab109531), Anti‐CD4 (ab133616), Anti‐CD8 (ab237709), Anti‐CD34 (ab81289), Anti‐CD68 (ab955), and Anti‐CCL3 (ab32609) were purchased from Abcam (Cambridge, UK). Anti‐CD20 (#48750) was purchased from Cell Signaling Technology (Danvers, MA, USA). Anti‐CCL21 (A1896) and Anti‐F4/80 (A23788) antibodies were purchased from Abclonal (Wuhan, China). Anti‐CXCL13 (NBP3‐07988) antibody was purchased from Novus Biologicals (Colorado, CA, USA). Type I collagenase (#SCR103) and Dispase II (#D4693) were purchased from Sigma‐Aldrich (St. Louis, MO, USA). The 40 µm Falcon Cell Strainer was purchased from Corning, NY, USA. The hematoxylin and eosin (#G1005) were purchased from Servicebio, Wuhan, China. The Opal 7‐Color Manual IHC Kit (#NEL811001KT) was purchased from Akoya Biosciences, USA.

### Patients

Three healthy samples and three pulpitis samples from different individuals were recruited for scRNA‐seq. Third molars in a healthy state (n = 5), or those with superficial caries (n = 5), moderate caries (n = 5), deep caries (n = 5), and pulpitis (n = 5) extracted due to therapeutic needs were collected for histological staining. The patients, aged 17–40 years, had teeth in good periodontal condition without any cracks. X‐ray films were obtained from all teeth before extraction to further confirm their physiological status. The study protocol was approved by the Medical Ethics Committee of the School and Hospital of Stomatology at Wuhan University (approval no.2020B75). All participants provided signed informed consent.

### Animals

Eight‐week‐old male C57BL/6 mice were acquired from the Hubei Provincial Laboratory Animal Research Center and maintained under SPF conditions. The occlusal surface of the upper left first molar was drilled using a #1/4 round bur and K files until the pulp was exposed, and the contralateral molar served as a healthy control. The exposed pulp remained open to the oral environment for 120 h (n = 6), after which the maxillae of the mice were dissected. All animal experiments received approval from the Animal Ethics Committee of Wuhan University (approval no. S07923120F).

### Tissue Collection and Immunohistochemical Staining

The extracted human teeth and isolated maxillae of mice were immediately fixed in 4% paraformaldehyde overnight. Ethylenediaminetetraacetic acid (EDTA; 10%) was used to decalcify the tissues, which were then dehydrated through a series of graded alcohol solutions and finally embedded in paraffin. A 5 µm‐thick slide was prepared. After deparaffinization, rehydration, and exposure to antigens, sections were incubated separately with Anti‐CD20, Anti‐CD68, Anti‐CCL21, Anti‐CD19, Anti‐F4/80, and Anti‐CD3 at 4 °C overnight and then processed according to the manufacturer's protocols. A diaminobenzidine reagent kit (Maxim, Fuzhou, China) and hematoxylin were used to stain the sections. Slides were scanned using SLIDEVIEW VS200 (Olympus, Tokyo, Japan). CD20, CD68, and CD3 expression in human dental pulp from various clinical states, as well as CD19, CD3, and F4/80 expression in mice with pulpitis, were quantified using histoscores at 40 ×  magnification, derived by multiplying the percentage of positively stained cells by their staining intensities.

### Hematoxylin and Eosin Staining

After deparaffinization, human tooth slides were rehydrated and then stained with hematoxylin and eosin (Service). Slides were scanned using the SLIDEVIEW VS200 (Olympus, Tokyo, Japan).

### Multiplex Immunofluorescence Staining

Multiplex immunofluorescence staining was performed with the Opal 7‐Color Manual IHC Kit (Akoya Biosciences, USA) according to the protocol recommended by the manufacturer. After deparaffinization and rehydration, tooth slides were subjected to antigen retrieval, blocking, primary antibody incubation, secondary antibody incubation, and fluorescent dye staining. The above steps were repeated until all primary antibodies had been incubated. DAPI was used to stain the cell nuclei. The primary antibodies used in this research were Anti‐CD20, Anti‐CD3, Anti‐CD4, Anti‐CD8, Anti‐CD68, Anti‐CD34, Anti‐CXCL13, and Anti‐CCL3. The slides were scanned using a Phenolmager HT (Akoya, MA, USA) with a 40 ×  objective lens. InForm software was used to split the fluorescence spectrum and obtain multiplex‐stained images.

### Cell Preparation for Single‐Cell RNA Sequencing

The method for obtaining cells from human dental pulp was consistent with our previous studies.^[^ ^19^ ^]^ Briefly, after the soft removal of the tooth, the dental pulp was immediately stored in the pre‐cooled tissue preservation solution. Samples were then cut into tiny pieces, digested with 4 mg mL^−1^ Dispase II and 3 mg mL^−1^ type I collagenase at 37 °C for 1 h, and filtered using the 40 µm Falcon Cell Strainer.

### Single‐Cell RNA Sequencing and Data Analysis

The procedure for single‐cell RNA sequencing and data analysis was consistent with that described in the previous study.^[^ ^19^ ^]^ Briefly, after obtaining the viability and concentration of the cell suspension, the single‐cell transcriptome capture was performed. Single‐cell transcriptome sequences captured using microbeads were collected into the cDNA library containing unique molecular identifiers (UMIs) and cell labels. All libraries were sequenced in PE150 mode (150‐bp paired‐end reads) using the Nextseq 2000 Illumina platform. The Raw sequencing data were processed using 10 × Genomics Cell Ranger pipeline (version 7.2.0) for demultiplexing and alignment to the hg38.

The Seurat in R package was used for subsequent data analysis, and the whole process included cell filtration, quality control, normalization and integration of the data, scaling, dimensionality reduction, cell clustering, and annotation.^[^ ^19^, ^29^, ^30^ ^]^ The ratio of mitochondria was not limited considering that anesthetic might damage the viability of cells. After scaled data according to UMI, Principal component analysis (PCA) was used to reduce the dimensionality of the datasets. Then, the singlets were clustered based on the first 20 principle components, which were chosen based on a JackStraw plot, a heatmap, and an elbow plot. These principal components were then further reduced in dimensionality using the UMAP. Transcriptional markers in each cluster were identified by using the FindAllMarkers function. Cell communication was assessed with CellPhoneDB, while the molecular functions of marker genes were analyzed through Metascape. Additionally, the regulons in each group were evaluated using IRIS3 following the recommended protocol.

### Statistical Analysis

All data were presented as mean ± standard deviation (SD). All experiments were repeated independently at least three times. Sample sizes for each experiment were listed in both the Experimental Section and the figure legends. Two‐tailed unpaired Student's *t‐*test was used to compare the statistical significance between the two groups. *P* value <0.05 was considered statistically significant. GraphPad Prism 8 software was used to analyze statistical data.

## Conflict of Interest

The authors declare no conflict of interest.

## Supporting information



Supporting Information

## Data Availability

The data that support the findings of this study are available from the corresponding author upon reasonable request.
