# Supplementary material for: Tertiary Lymphoid Structure in Dental Pulp: The Role in Combating Bacterial Infections
Source: Adv Sci (Weinh). 2024 Oct 28;12(1):2406684. doi: 10.1002/advs.202406684 (PMC11714202; doi:10.1002/advs.202406684)
Supplement: Supplementary file 1 — Supporting Information [file ADVS-12-2406684-s001.docx]

Supporting Information

**Tertiary Lymphoid Structure in Dental Pulp: The Role in Combating Bacterial Infections**

*Ruiqi Li*, *Fan Gu*, *Linlin Peng*, *Tingting Huan*, *Zhuo Zhou, Yaling Song*, *Jinmei He*, *Kaili Ye*, *Yao Sun*, *Tiejun Li*, *Miao He*^*^, *Zhuan Bian*^*^ ,*Wei Yin*^*^

**
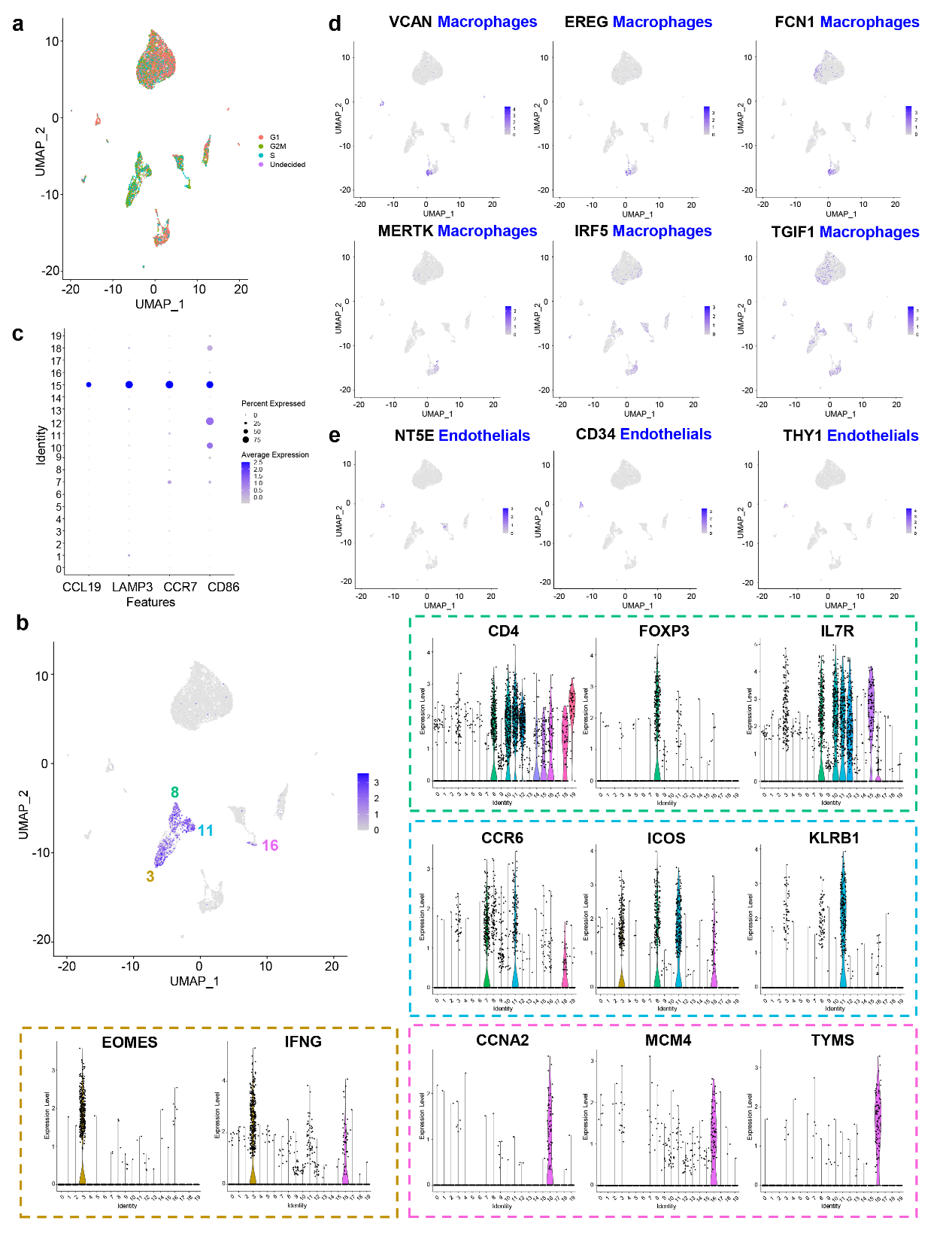
**

**Figure S1.** Cells in dental pulp with inflammation. a) The cell cycle of cells in inflammatory dental pulp. b) The markers of four clusters (cytotoxic T cells in cluster 3, Treg cells in cluster 8, Th cells in cluster 11, and Trm cells in cluster 16) in inflammatory pulp. Markers in brown, green, blue or magenta box represented cluster 3, 8, 11 or 16 respectively. c) The makers of DCs in cluster 15 (CCL19, LAMP3, CCR7, and CD86). d) Signature genes in M2 macrophages. e) The hematopoietic stem cell marker and endothelial markers in cluster 13 (endothelial cells).


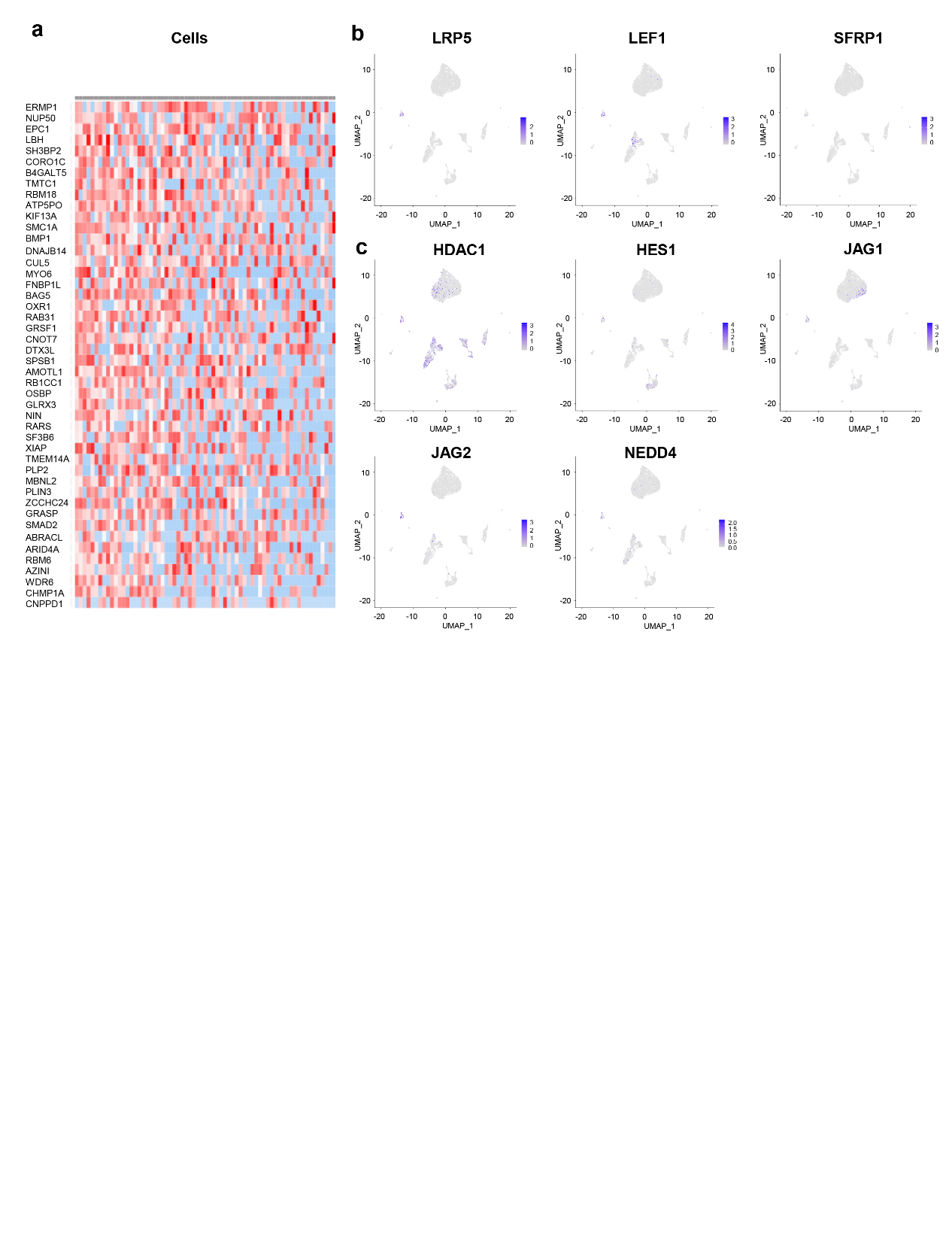


**Figure S2.** Cells in dental pulp with inflammation. a) The regulons in endothelial cells. b-c) Signature markers of cluster 13 (endothelial cells) in Wnt (LRP5, LEF1, and SFRP1) and Notch (HDAC1, HES1, JAG1, JAG2, and NEDD4) signaling pathways.

**
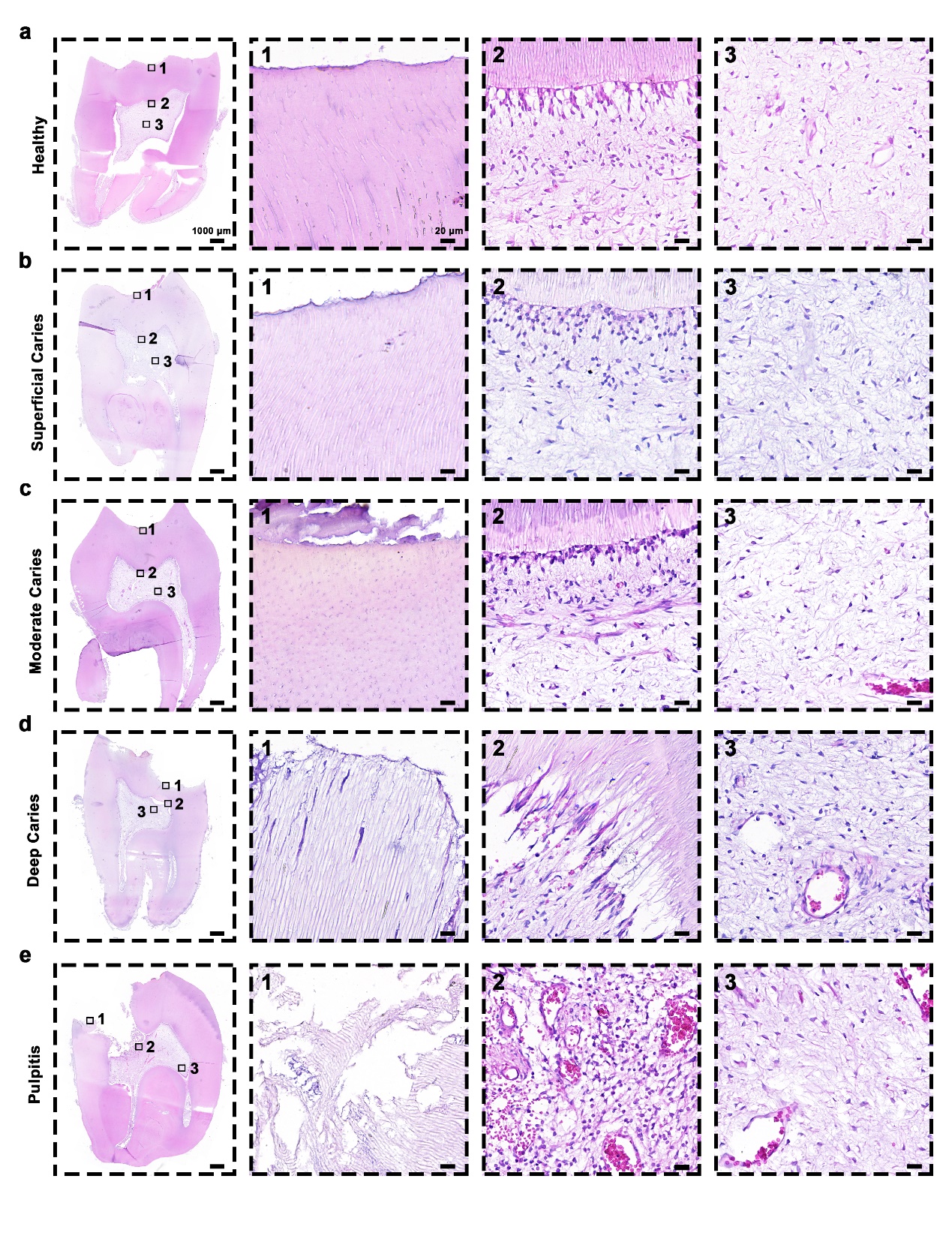
**

**Figure S3.** H&E staining images of dental pulp. a) H&E staining of healthy dental pulp. b) H&E staining of dental pulp with superficial caries. c) H&E staining of dental pulp with moderate caries. d) H&E staining of dental pulp with deep caries. e) H&E staining of dental pulp with pulpitis. The dotted boxes 1, 2, and 3 displayed magnified images of the superficial layer of dentin, the dentin-pulp demarcation, and the dental pulp, respectively. Scale bars = 1000 μm or 20 μm.


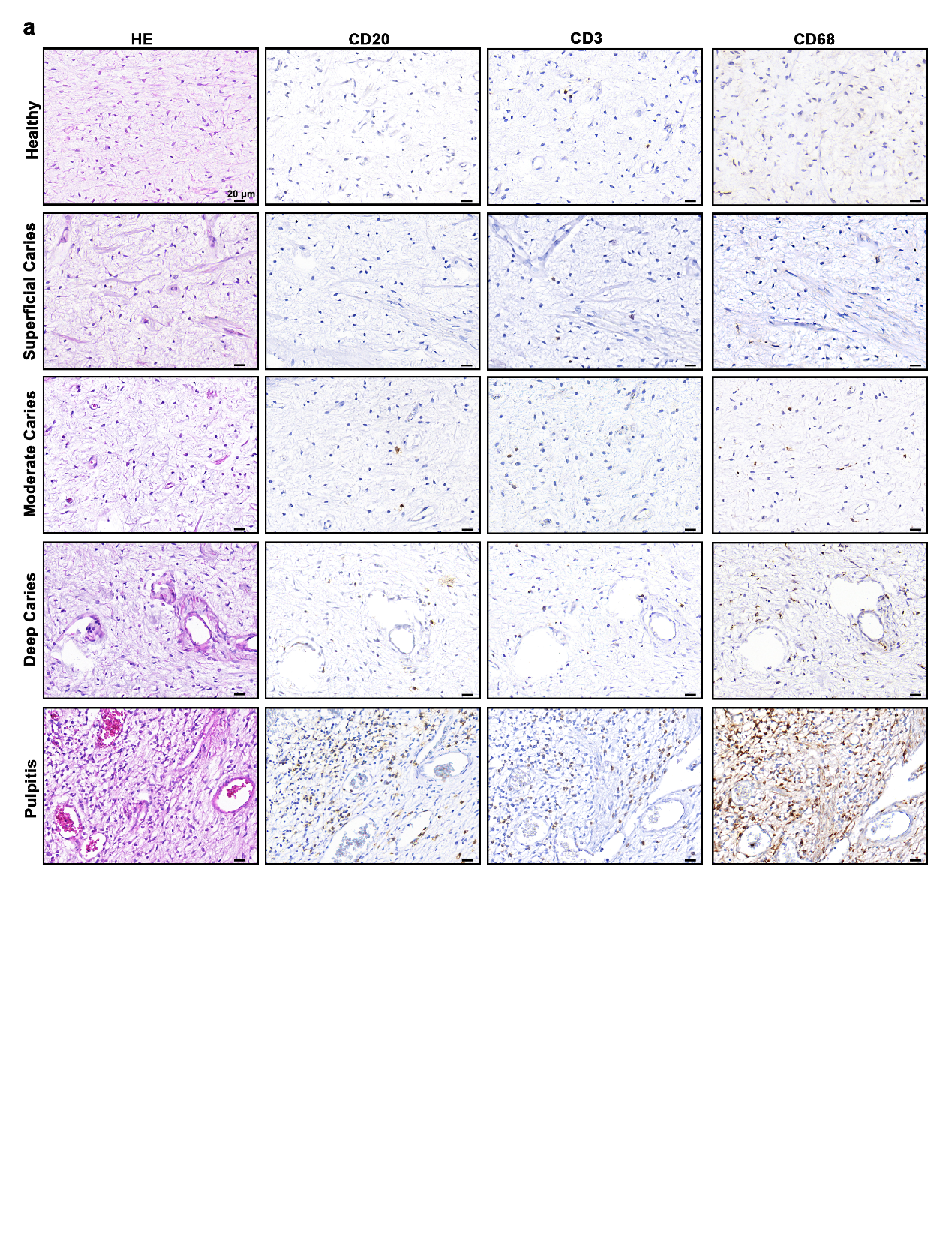


**Figure S4.** Expression of immune cells in TLS. a) Representative H&E and IHC staining images of CD20, CD3, and CD68 in serial sections of human dental pulp across different clinical states (healthy dental pulp, and dental pulp with superficial caries, moderate caries, deep caries, and pulpitis). Scale bar = 20 μm.


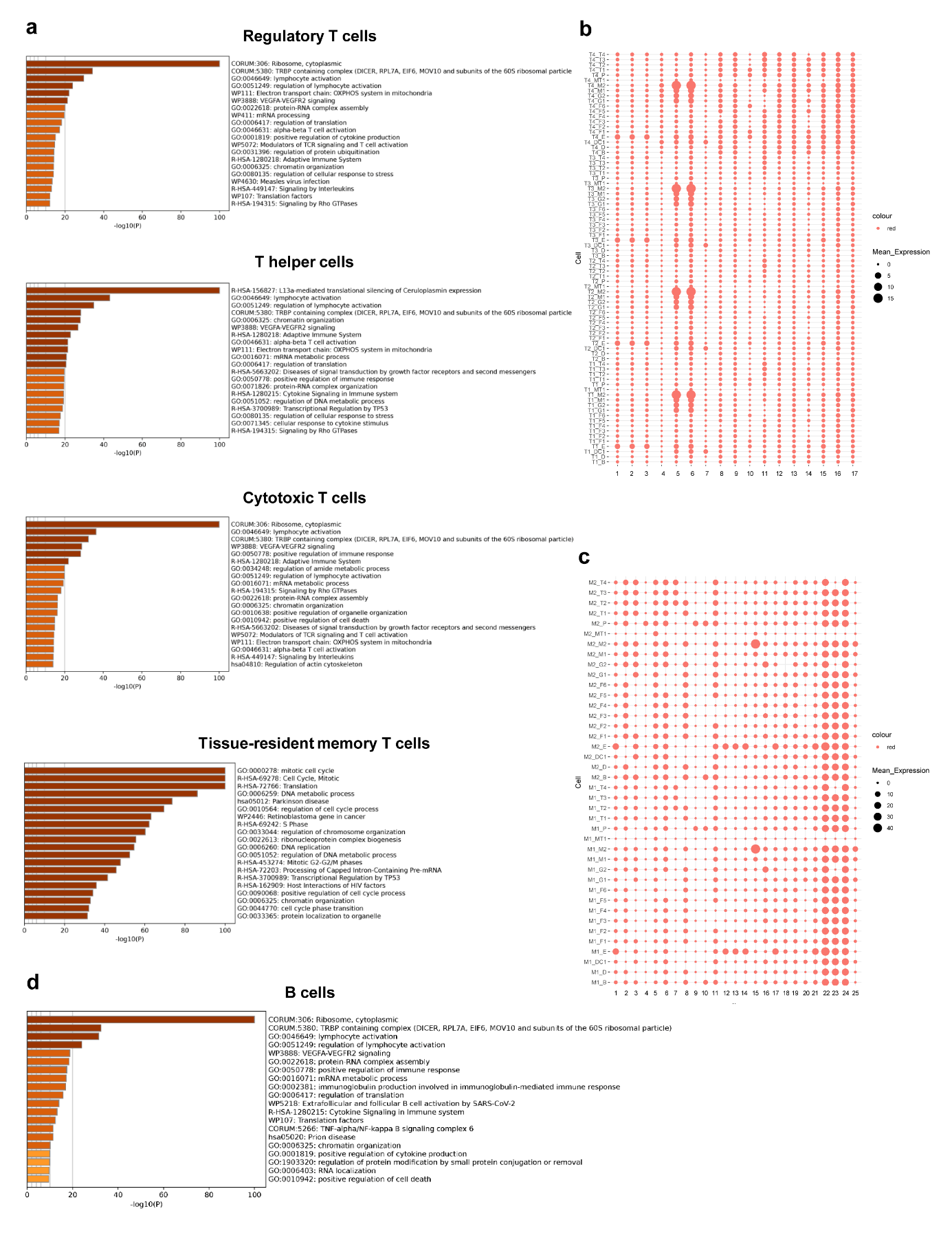


**Figure S5.** Interactions of immune cells in TLS. a) Molecular functions of the active T cells in inflamed dental pulp. b) Predicted interactions between T cells and other cell types in the inflamed pulp. c) Predicted interactions between macrophages and other cells. d) Molecular functions of unique signature genes in B cells.
